# The History of Appendicitis

**Published:** 1904-07

**Authors:** 


### THE HISTORY OF APPENDICITIS.

Dr. Howard A. Kelly, of Baltimore, has been dipping into the history of appendicitis. From an address delivered by him some time ago in Paris we learn that Mestiver, in 1759, reported a characteristic case of appendicitis. There was a swelling on the right side of the umbilicus. This was incised, and about a pint of fetid pus escaped. At the necropsy there was found a pin in the appendix. It was crusted over and eroded. This had clearly set up the disease. From the same reference in the *British Medical Journal* we learn that in 1776 Joubert Lamotte published the report of a case, the patient having died with marked tympanites. At the autopsy a concretion was found in the appendix and some cherries in the cecum. This is the first instance of a fecal calculus. In 1824 Layer-Villarmay published a paper on "Observations to Serve for the History of Inflammation of the Cecal Appendix." He relates two cases with necropsy. He pointed out the importance of inflammation in the appendix. In 1827 Melier published an article on the subject and related some new cases. He speaks of the nature of the lesions, and even thought surgical treatment might be justified. He was frowned down by Duputren, then the leading surgeon of France, and Melier's brilliant suggestion came to nothing for many years.—*Canada Lancet.*

